# Expression of tumor-associated calcium signal transducer 2 in patients with salivary adenoid cystic carcinoma: Correlation with clinicopathological features and prognosis

**DOI:** 10.3892/ol.2014.2400

**Published:** 2014-07-31

**Authors:** YICHAO XIA, BO LI, NING GAO, HUI XIA, YI MEN, YING LIU, ZHE LIU, QIANMING CHEN, LONGJIANG LI

**Affiliations:** 1 Department of Head and Neck Oncology Surgery, West China College of Stomatology, Sichuan University, Chengdu, Sichuan 610041, P.R. China; 2 Department of Pathology, State Key Laboratory of Oral Diseases, West China College of Stomatology, Sichuan University, Chengdu, Sichuan 610041, P.R. China

**Keywords:** salivary adenoid cystic carcinoma, TACSTD2, prognosis, clinicopathological feature

## Abstract

Salivary adenoid cystic carcinoma (SACC) is a common salivary malignancy. The current treatment option for SACC is complete surgical excision with postoperative radiotherapy. The prognosis remains unsatisfactory, due to frequent local recurrence and distant metastases that directly reduce the overall survival time. Previous studies have shown that overexpression of tumor-associated calcium signal transducer 2 (TACSTD2) is associated with poor prognosis in various human epithelial cancers. The expression of TACSTD2 in SACC is currently unknown. The present study therefore aimed to retrospectively investigate TACSTD2 protein expression by immunohistochemistry on paraffin-embedded primary tumor tissue samples from a series of consecutive SACC patients (n=81). The correlation of TACSTD2 expression with clinicopathological variables was evaluated using either the Kruskal-Wallis or Mann-Whitney statistical tests. The survival curves were plotted using the Kaplan-Meier method. The parameters of prognostic significance found by univariate analysis were verified in a multivariate Cox regression model. Overexpression of TACSTD2 was detected in 35/81 (44%) SACC patients and was significantly associated with a decreased overall survival (P<0.01). Univariate analysis showed that TACSTD2 overexpression was correlated with TNM stage (P=0.020), local recurrence (P=0.002) and distant metastasis (P=0.001). Multivariate analyses further revealed that TACSTD2 may be an independent prognostic indicator. In conclusion, TACTSD2 could be recognized as an independent prognostic indicator for SACC. Gene therapy targeting TACSTD2 may be a possible treatment approach for patients with SACC overexpressing this cell-surface marker.

## Introduction

Adenoid cystic carcinoma (ACC) is the second most frequent salivary malignancy, accounting for 10–18% of all salivary malignancies (1,2). Clinical characteristics for ACC include invasive local growth, frequent local recurrence and distant metastasis. The conventional treatment for ACC is radical surgery with postoperative radiotherapy. Despite a relatively high short-term survival rate, the likelihood of recurrence and metastasis is high. The 5- and 10-year overall survival rates for patients without distant metastasis are 85.4 and 67.4%, respectively. The 5- and 10-year overall survival rates for patients with distant metastasis are 69.1 and 45.7%, respectively (3). Further investigation of the possible biomarkers involved in the progression and metastasis of salivary ACC (SACC) may help identify novel therapeutic strategies and improve the long-term outcome of the disease.

The tumor-associated calcium signal transducer (TACSTD) gene family consists of two highly related genes, TACSTD2 and TACSTD1. TACSTD1 (also known as Trop1 or EpCAM) was originally identified as a marker for epithelial carcinomas due to aberrant expression in various tumors (4). TACSTD2 (also known as Trop2, GA733-1, M1S1 or EGP-1) is a 36-kDa type-I transmembrane glycoprotein that was originally identified in human placental trophoblasts, and may function to transduce an intracellular calcium signal (5,6). TACSTD1 and 2 share ~50% sequence identity and both contain a thyroglobulin repeat domain region (7). The present study speculated that TACSTD2 may also function in tumor formation and development. It has been previously shown that the cytoplasmic tail of TACSTD2 is essential for signaling, and phosphorylation of serine 303 regulated by protein kinase C can control the tumor growth stimulatory capacity of TACSTD2 (8,9).

TACSTD2 overexpression has been reported in numerous human cancers, such as colorectal, ovarian, pancreatic, cervical, gastric, bile duct cancer and squamous cell carcinoma of the oral cavity, as compared with the corresponding normal tissue, and the expression level has been shown to correlate with the poor patient prognosis (10–16). The expression and function of TACSTD2 in human SACC has not been previously reported.

The present study aimed to retrospectively investigate TACSTD2 antigen expression by immunohistochemistry, in paraffin-embedded primary tumor tissue samples from a series of consecutive SACC patients (n=81). The correlation of TACSTD2 expression with clinicopathological variables and survival analysis was evaluated.

## Materials and methods

### Patient material and tumor samples

The present study was approved by the Institutional Ethics Committee of Sichuan University (Chengdu, China) and consent was obtained from the patients. Paraffin-embedded tissue specimens, including 81 SACCs, were randomly selected from the West China College of Stomatology, Sichuan University between January 1991 and December 1996. All of these recruited patients underwent radical surgery without preoperative chemotherapy or radiotherapy, and the collected samples were reconfirmed by two pathologists. Data from the patient follow-up and clinicopathological characteristics were collected from the database of West China College of Stomatology and from telephone interviews. The recurrence and metastasis during the follow-up period was confirmed by radiography and pathology biopsy. Of the 81 patients, there were 37 males and 44 females, and the median age was 47 (range, 20–81 years). The overall survival (OS) was calculated from the first radical surgery until death due to any cause. The patients who had survived at the time of the last follow-up visit were lost to further follow-up. The details of the patient data are listed in Table I. Five normal salivary gland tissue samples were selected to be used as controls. The clinical staging of the patients was determined according to the International Union against Cancer TNM Classification of Malignant Tumors (17). The histologic subtypes of the samples were decided on the basis of the World Health Organization’s histologic classification of salivary gland tumors (18).

### Immunohistochemical staining

Formalin-fixed, paraffin-embedded tissues were consecutively cut into 4-μm sections and transferred onto the silanized glass slides. The slides were dewaxed in xylene and rehydrated through a graded alcohol series. Endogenous peroxidase activity was blocked with 3% hydrogen peroxide solution for 20 min at room temperature. The slides were heated to high-temperature in 0.1 M citrate buffer (pH 6.0) for 4 min to repair the antigen and then blocked with 1% bovine serum albumen Tris-HCl buffer. The slides were incubated overnight at 4°C with a polyclonal goat antibody against the human TACSTD2 extracellular domain (R&D Systems, Inc., Minneapolis, MN, USA) at a 1:100 dilution. A biotinylated polyclonal rabbit anti-goat antibody (Vector Labs, Burlingame, CA, USA) at a 1:200 dilution was applied to reveal the primary antibody. Horseradish peroxidase-streptavidin (Dako, Glostrup, Denmark) and diaminobenzidine were used as chromogens. All the sections were counter-stained with hematoxylin and then dehydrated and mounted. Negative controls were sections that had been incubated with phosphate-buffered saline instead of the primary antibody.

### Immunohistochemical evaluation

The level of immunoreactivity was separately evaluated by two independent pathologists without any prior information of the clinical data. Samples with disagreement in the immunohistochemical score were rescored under the discussion of the two observers. A scoring method according to the percentage of positive tumor cells and the intensity of the staining was applied. For intensity: 0, negative; 1, weak; 2, moderate; 3, strong; for the percentage: scored 0, <1%; 1, ≥1% to <10%; 2, ≥10% to <50%; 3, ≥50%. The final score was obtained by multiplying the two scores: score 0, negative; scores 1 and 2, weakly positive; scores 3 and 4, moderately positive; scores 6 and 9, strongly positive.

### Statistical analysis

The correlation of TACSTD2 expression with clinicopathological variables was evaluated by Pearson’s χ^2^ test or Fisher’s exact test. The survival curves were plotted using the Kaplan-Meier method, and the significance of differences in the OS rate was tested by the log-rank test. The parameters of prognostic significance found by univariate analyses were verified in a multivariate Cox proportional-hazards regression model. P<0.05 was considered to indicate a statistically significant difference. Statistical analysis was performed using SPSS statistical software, version 18.0 (SPSS, Inc., Chicago, IL).

## Results

### TACSTD2 expression in SACC specimens and association with clinicopathological parameters

**Figure 1:**
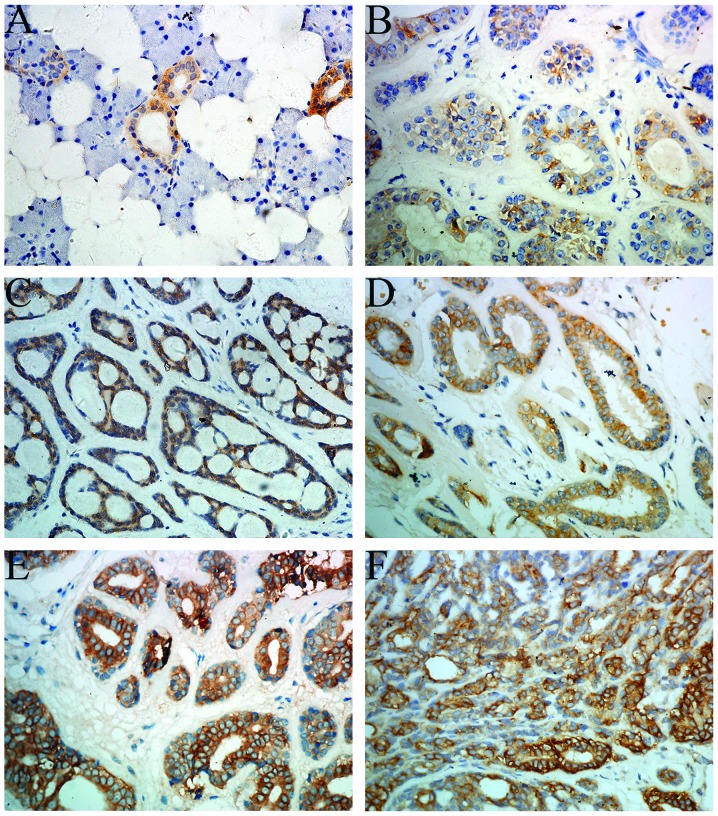
Expression of TACSTD2 in SACC (magnification, ×400). (A) The expression of TACSTD2 in the normal salivary gland. (B) Weak staining of TACSTD2 was observed in a tubular subtype of SACC. (C and D) Moderate staining of TACSTD2 was detected in cribriform and tubular subtypes of SACC, respectively. (E and F) Strong staining of TACSTD2 was observed in tubular and solid subtypes of SACC, respectively. TACSTD2, tumor-associated calcium signal transducer 2; SACC, salivary adenoid cystic carcinoma.

Expression of TACSTD2 in normal salivary gland tissue was not detected except in some ductal epithelial cells (Fig. 1A). The positive staining of TACSTD2 primarily occurred on the cell membrane. Positive expression of TACSTD2 was noted in 63/81 cases (Fig. 1B–F). By subgroup analysis, negative expression was observed in 18 (22.2%) cases, weak expression in 27 (33.3%) cases, moderate expression in 20 (24.7%) cases and strong expression in 16 (19.7%) cases. The correlations between TACSTD2 expression and clinicopathological variables are listed in Table I. The statistical analysis identified that no significant difference was observed between the TACSTD2 expression level and clinical factors of age, gender, histological subtype and perineural invasion; however, TNM stage (P=0.020), local recurrence (P=0.002) and distant metastasis (P=0.001) were noted to be associated with a higher expression of TACSTD2.

### Survival analysis

**Figure 2:**
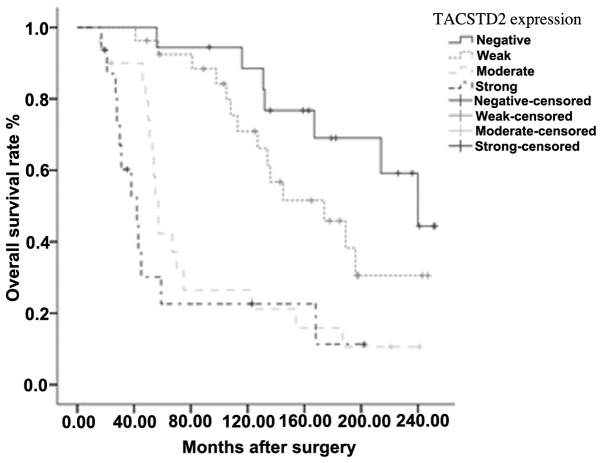
Kaplan-Meier survival curves for cumulative survival rate of patients with salivary adenoid cystic carcinoma according to TACSTD2 expression (P<0.001, comparison between the survival analysis results of the negative, weakly positive, moderately positive and strongly positive TACSTD2 expression). TACSTD2, tumor-associated calcium signal transducer 2.

The Kaplan-Meier method was used to perform a univariate analysis. A survival curve, according to the TACSTD2 expression level, was plotted. The OS rates decreased with increasing TACSTD2 expression level (Fig. 2). The variables with a significant difference, as tested by log-rank analysis, were histological subtype (P<0.001), TNM stage (P=0.003), local recurrence (P<0.001), distant metastasis (P<0.001) and TACSTD2 expression (P<0.001). The statistical data are detailed in Table II. To further assess the impact of TACSTD2 expression on survival, all the statistically significant variables, including TACSTD2 expression, were subjected to the Cox proportional-hazards regression model. TACSTD2, histological subtype and distant metastasis were identified to be independent prognostic indicators for overall survival (Table II).

## Discussion

Despite numerous advances in the diagnosis and treatment of SACC over the past 20 years, a high frequency of local recurrence and distant metastasis has remained. Treatments based on conventional clinicopathological parameters are valuable but are not satisfactory in improving the OS. Specific gene-targeting therapy may be an alternative option to consider for patients with SACC. Investigations of cell surface markers expressed in tumor cells could be a possible method to identify novel gene targets.

TACSTD2, formed by the retropositioning of EpCAM via an mRNA intermediate, is located on chromosome 1p32 and encodes an intronless gene product (19). TACSTD2 protein is a type I membrane protein conducting calcium signaling (5). Retrospective studies have found that overexpression of TACSTD2 predicts poor prognosis in the majority of human cancers. Serological identification of TACSTD2 in patients with esophageal squamous cell carcinoma suggested that the TACSTD2 antigen may be a valuable serum tumor marker (20). The bicistronic cyclin D1-TACSTD2 mRNA was isolated and was shown to be a potent oncogene both *in vitro* and *in vivo* 21). The expression of the chimeric mRNA was observed to improve the stability of cyclin D1 and enhance the proliferation of the expressing cells (21). The signaling mechanism of TACSTD2 involved in tumor pathogenesis was first explained by activating the extracellular signal regulated kinase (ERK)/mitogen-activated protein kinase pathway, and cyclin D1 was activated as an important downstream factor of the pathway (22). A detailed TACSTD2 signaling network in cancer growth was further elucidated by Guerra *et al* (21); TACSTD2 upregulation was shown to subsequently drive the expression and activation of CREB1, Jun, NF-kB, Rb, STAT1 and STAT3 through induction of the cyclin D1 and ERK/MEK pathways (8). Chemoresistance has been shown in cell lines overexpressing cyclin D1, which suggested that cyclin D1 may contribute to chemoresistance (23). Recent studies have shown that TACSTD2 has a critical role in the metastasis of prostate cancers by modulating β1 integrin function, and activation of PAK4 induced by TACSTD2 was observed in the experiment (24,25).

Immunotherapeutic agents against EpCAM, the analog of TACSTD2, have produced promising results (26,27). Furthermore, hRS7, a human monoclonal anti-TACSTD2 antibody has been used in endometrial endometrioid carcinoma (EEC). Cases of EEC overexpressing TACSTD2 were shown to be highly sensitive to hRS7-mediated cytotoxicity *in vitro* (28). Similar results were achieved in uterine and ovarian carcinosarcomas (29,30). In addition, hRS7 also showed a significant therapeutic advantage in an *in vivo* breast cancer model (31,32).

To the best of our knowledge, the present study is the first to analyze the expression, prognostic value and clinical significance of TACSTD2 in 81 patients with SACC. The results were consistent with the previously known functions of TACSTD2 in tumor development and metastasis. The expression of TACSTD2 was significantly associated with tumor TNM stage (P=0.020), local recurrence (P=0.002) and distant metastasis (P=0.001). By multivariate analysis, TACSTD2 was shown to be an independent prognostic indicator of SACC; however, for the inherent retrospective analyses limitations, overexpression of TACSTD2 as the prognostic indicator needs to be validated in larger prospective studies. In conclusion, TACSTD2 is a surface antigen that is overexpressed in various epithelial cancers and may be a novel therapeutic target.

## Figures and Tables

**Table I:** Expression of TACSTD2 in salivary adenoid cystic carcinoma and its association with clinicopathological variables.

		TACSTD2 expression				
			
	n	N, n (%)	W, n (%)	M, n (%)	S, n (%)	P-value
Gender	81	18 (22.2)	27 (33.3)	20 (24.7)	16 (19.7)	0.582^a^
Male	37	10 (27.0)	11 (29.7)	9 (24.3)	7 (18.9)	
Female	44	8 (18.2)	16 (36.4)	11 (25.0)	9 (20.5)	
Age (years)						0.328^a^
≤50	46	12 (26.1)	16 (34.8)	9 (19.6)	9 (19.6)	
>50	35	6 (17.1)	11 (31.4)	11 (31.4)	7 (19.8)	
Histological subtype						0.289^b^
Tubular	32	6 (18.8)	14 (43.8)	7 (21.9)	5 (15.6)	
Cribriform	28	9 (32.1)	8 (28.6)	5 (17.9)	6 (21.4)	
Solid	21	3 (14.3)	5 (23.8)	8 (38.1)	5 (23.8)	
TNM Stage						0.020^a^
I+II	32	13 (40.6)	8 (25.0)	6 (18.8)	5 (15.6)	
III+IV	49	5 (10.2)	19 (38.8)	14 (28.6)	11 (22.4)	
Perineural invasion						0.053^a^
Negative	41	13 (34.2)	11 (28.9)	8 (21.1)	6 (15.8)	
Positive	40	5 (11.6)	16 (37.2)	12 (27.9)	10 (23.3)	
Local recurrence						0.002^a^
Negative	35	12 (34.3)	14 (40.0)	6 (17.1)	3 (8.6)	
Positive	46	6 (13.0)	13 (28.3)	14 (30.4)	13 (28.3)	
Distant metastasis						0.001^a^
Negative	52	15 (28.8)	20 (38.5)	12 (23.1)	5 (9.6)	
Positive	29	3 (10.3)	7 (24.1)	8 (27.6)	11 (37.9)	

TACSTD2 expression was scored according to the percentage of positive tumor cells and the intensity of staining. N, negative; W, weakly positive; M, moderately positive; S, strongly positive.

a Mann-Whitney and

b Kruskal-Wallis test.

TACSTD2, tumor-associated calcium signal transducer 2.

**Table II:** Univariate and multivariate analysis of clinicopathological variables and Trop2 expression in relation to overall survival in patients with salivary adenoid cystic carcinoma.

	Univariate analysis	Multivariate analysis		
		
Risk factors	P-value	HR	95% CI	P-value
Overall survival				
Age (≤50/>50)	0.134	Not included in model		
Gender (Male/Female)	0.549	Not included in model		
Histological subtype (C, S/T)	<0.001	2.610	1.159–5.876	0.020
TNM Stage (I+II/III+IV)	0.003	1.802	0.871–3.731	0.112
Perineural invasion (N/P)	0.180	Not included in model		
Local recurrence (N/P)	<0.001	1.427	0.726–2.807	0.302
Distant metastasis (N/P)	<0.001	3.163	1.379–7.258	0.007
TACSTD2 Expression (N/W/M/S)	<0.001	<0.001^b^		
TACSTD2 (W/N)		1.521	0.584–3.960	0.390
TACSTD2 (M/N)		3.791	1.436–10.010	0.007
TACSTD2 (S/N)		11.193	3.953–31.690	<0.001

HR, hazard ratio; CI, confidence interval; N, negative; P, positive; T, tubular; C, cribriform; S, solid; W, weakly positive; M, moderately positive; S, strongly positive; HR, hazard ratio; TACSTD2, tumor-associated calcium signal transducer 2.
